# Supplementary material for: A quantum-mechanical framework for million-atom scale biological systems
Source: Commun Chem. 2026 Apr 29;9:170. doi: 10.1038/s42004-026-02038-y (PMC13128919; doi:10.1038/s42004-026-02038-y)
Supplement: Supplementary file 2 — Supporting Information [file 42004_2026_2038_MOESM2_ESM.pdf]

Supplementary Information

A Quantum-Mechanical Framework for Million-Atom Scale Biological Systems

Luc Wieners[1] & Martin E. Garcia[1]*

Affiliation
[1]Institute of Physics, University of Kassel, Heinrich-Plett-Straße 40, 34132 Kassel, Germany
*Correspondence to: garcia@physik.uni-kassel.de

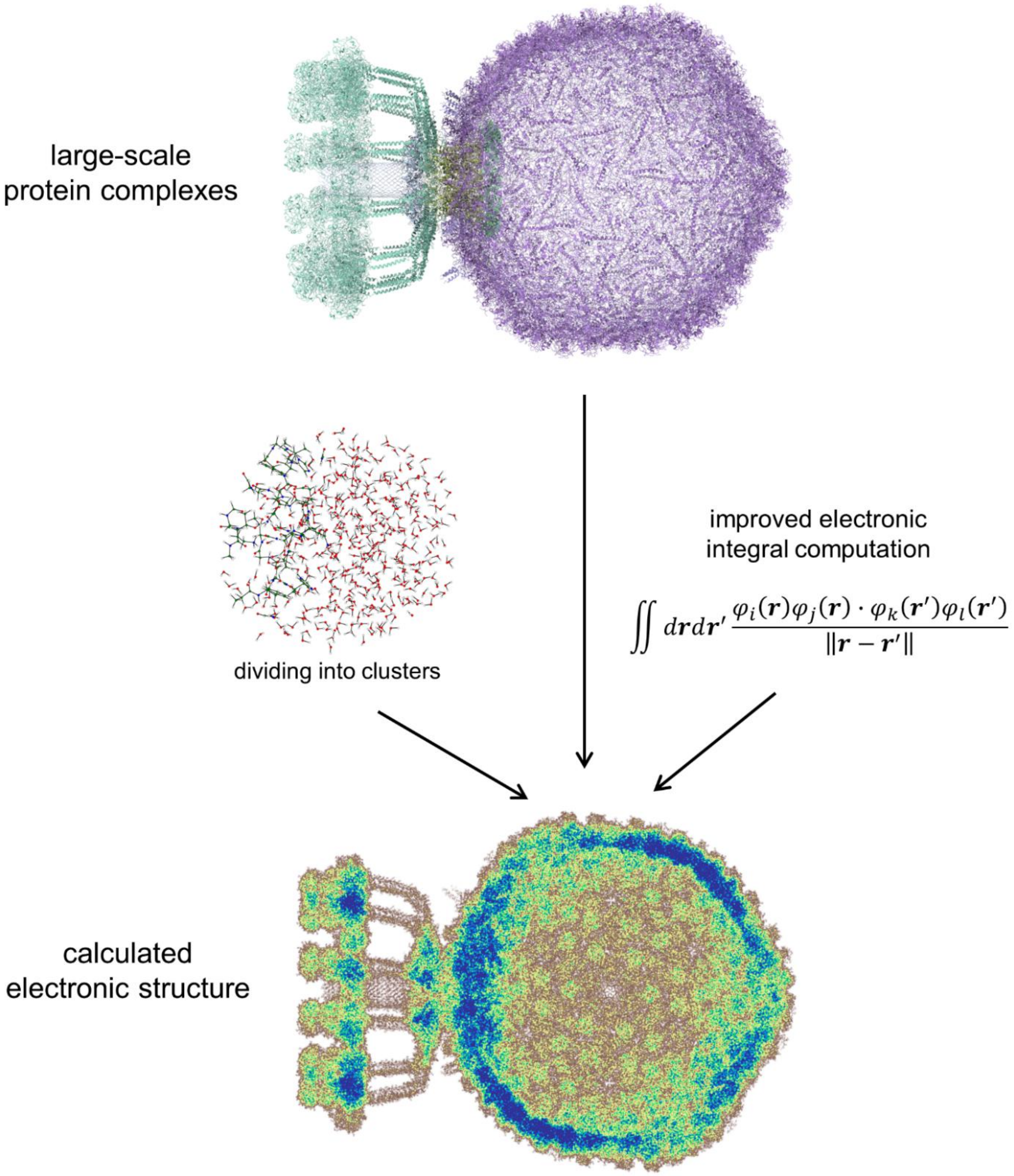

Supplementary Figure 6: Graphical abstract for large-scale quantum-mechanical calculations. Accessing the electronic structure (right) of large-scale biological structures as the bacteriophage P68[1] (left) is made possible by dividing the structure into clusters and employing an improved algorithm for electronic integral computation.

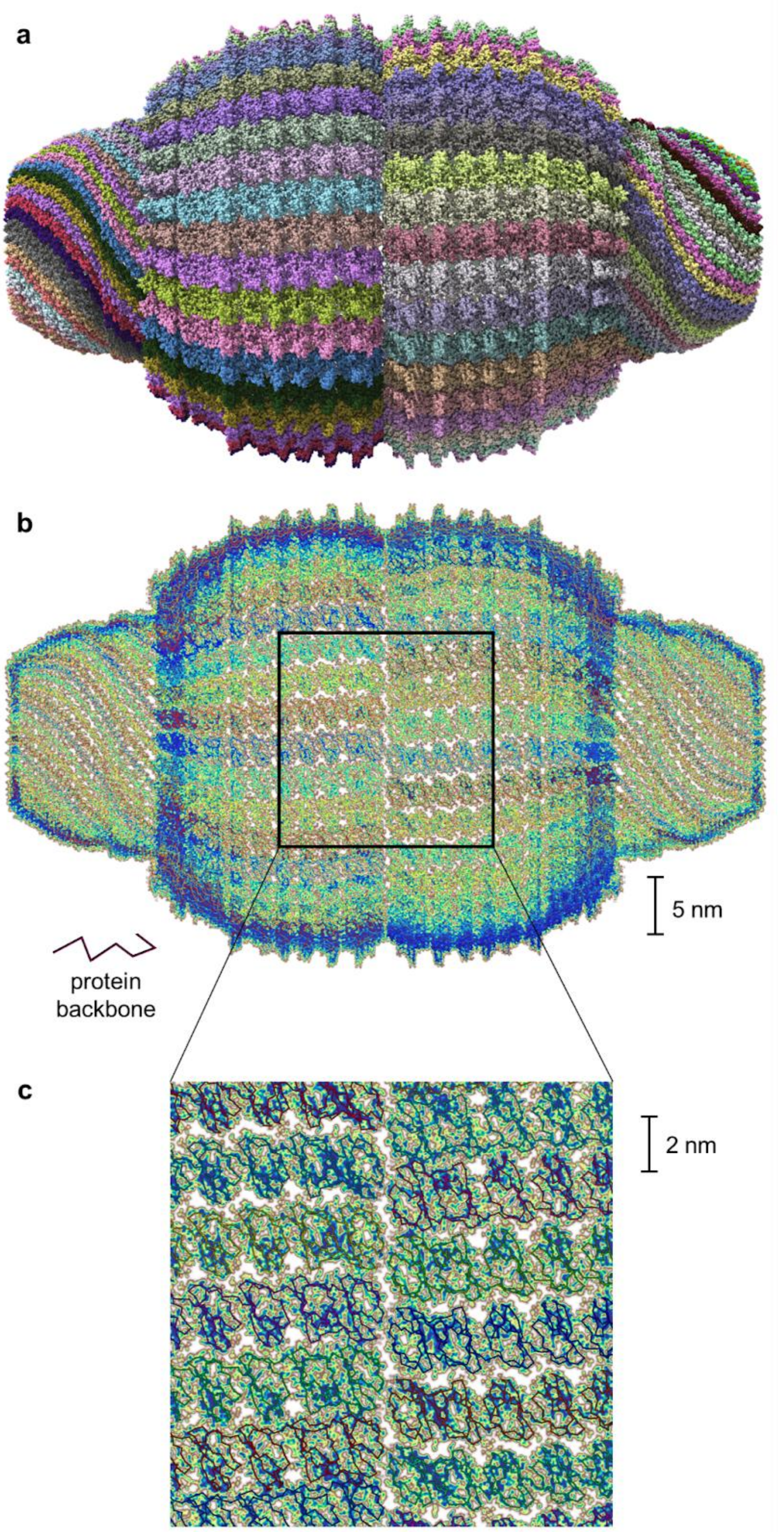

Supplementary Figure 7: Calculated electronic density of the vault protein complex. Detailed depiction of the electronic density of the vault protein complex PDB 7PKR[39], computed using the Hartree-Fock method presented in this work. **a**, Atomistic representation of the complete molecule. **b**, Projection of the electronic density of one half on the vault structure along the x-axis. **c**, Close-up view of the region highlighted in **b**. The protein backbone is displayed as coloured lines, with brown indicating regions of low and blue of high electronic density. For clarity, the electronic density of the solvent is omitted and only contributions from valence basis functions are shown.

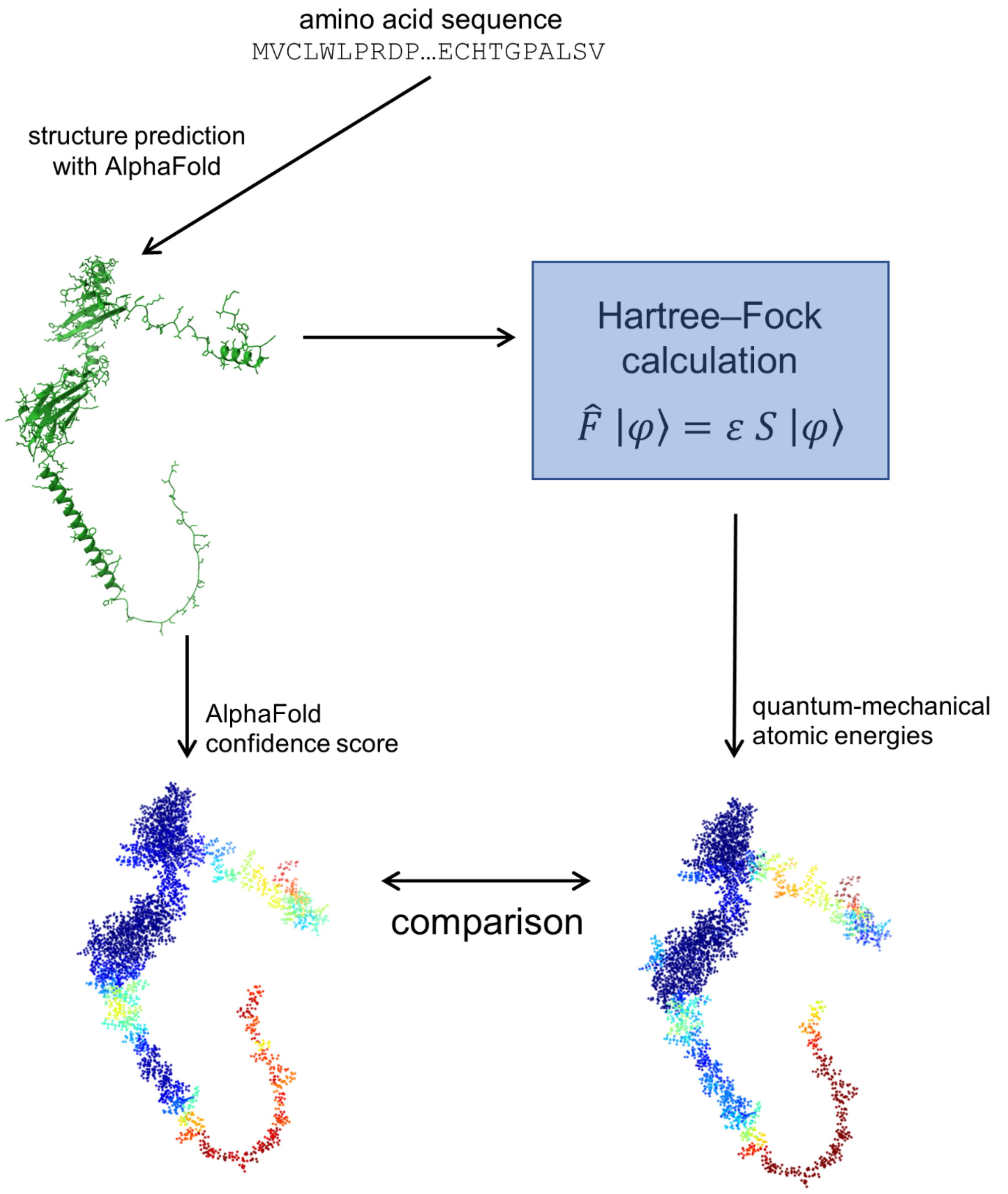

Supplementary Figure 8: Comparison of quantum energies and pLDDT scores. Graphical abstract illustrating one of the main findings of this work: the correlation between AlphaFold's confidence scores and quantum-mechanical atomic energies. A structure prediction from AlphaFold (based on a known amino acid sequence) is analysed using Hartree–Fock calculations to determine atomic energies. These energies are then compared with the pLDDT scores, which are a direct output from AlphaFold. The visualisation of the protein structure PDB AF_AFA0A023FF81F1 was generated using ChimeraX[34] with data from rcsb.org[47].

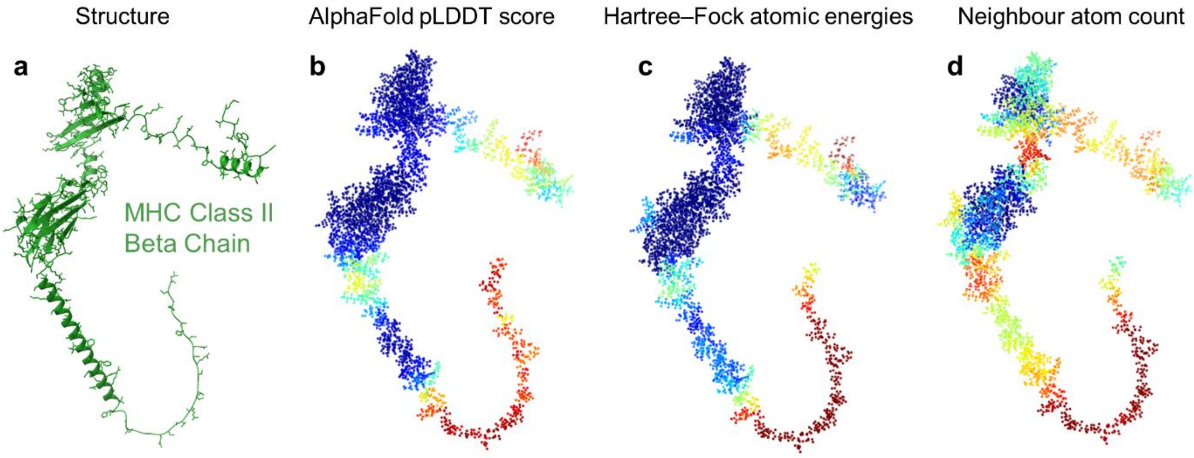

Supplementary Figure 9: Atomic energies versus neighbouring atom count. For the predicted protein structure PDB AF_AFA0A023IKK2F1[47] (**a**), the structure evaluation method using atomic energies from Hartree–Fock is compared with an approach that uses the number of neighbouring atoms as a metric. Higher local atom density is typically associated with more accurate predictions, resulting in a loose correlation between the neighbouring atom count (**b**) and AlphaFold's pLDDT scores (**d**). However, the evaluation of protein structures with Hartree–Fock atomic energies (**c**) provides a more accurate assessment. For example, the alpha helix at the bottom left of the structure is correctly assigned high confidence by the quantum approach but not by the neighbour count method. Similarly, the linker between the two top-left domains is identified as stable by the Hartree–Fock energies but misclassified as a weak region by the atom density method.

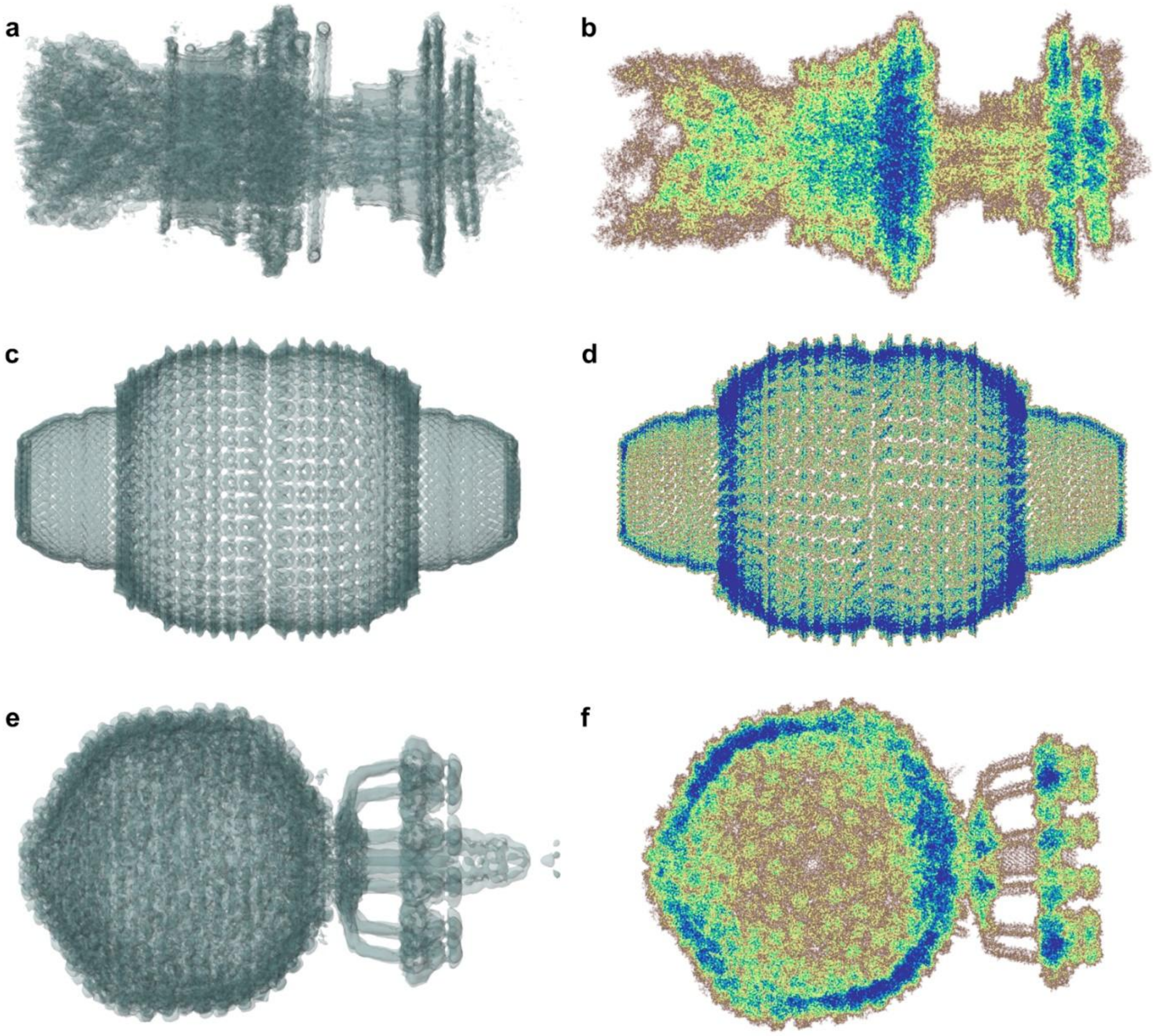

Supplementary Figure 10: Cryo-EM and computed densities. Comparison of electronic densities determined via cryogenic electron microscopy (**a**,**c**,**e**) with quantum-mechanically computed electronic densities (**b**,**d**,**f**) for a flagellar motor (PDB 8WL2)[38], a vault protein complex (PDB 7PKR)[39] and a bacteriophage (PDB 6Q3G)[1]. **a**,**c**,**e** were visualised using the Mol* Viewer[96] from rcsb.org[47].

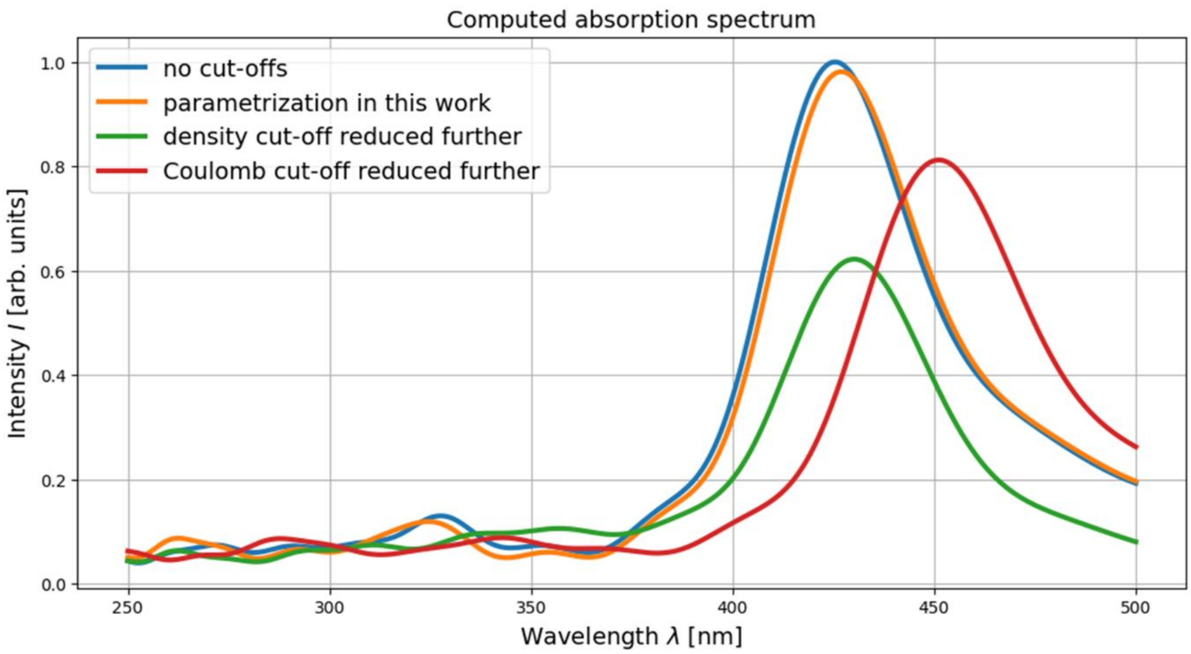

Supplementary Figure 11: Absorption spectra for beta-carotene with different parametrizations. The computed absorption spectrum of the molecule beta-carotene ($C_{40}H_{56}$) using RTHF. Four different configurations are shown: no density cut-off and no Coulomb cut-off (blue), density cut-off 10^{-6} and Coulomb cut-off 10 Å (orange), density cut-off 10^{-3} and Coulomb cut-off 10 Å (green), density cut-off 10^{-6} and Coulomb cut-off 6 Å (red). The second parametrization is used for the UV/Vis spectra in this work and shows little deviation from the calculation with no cut-offs.

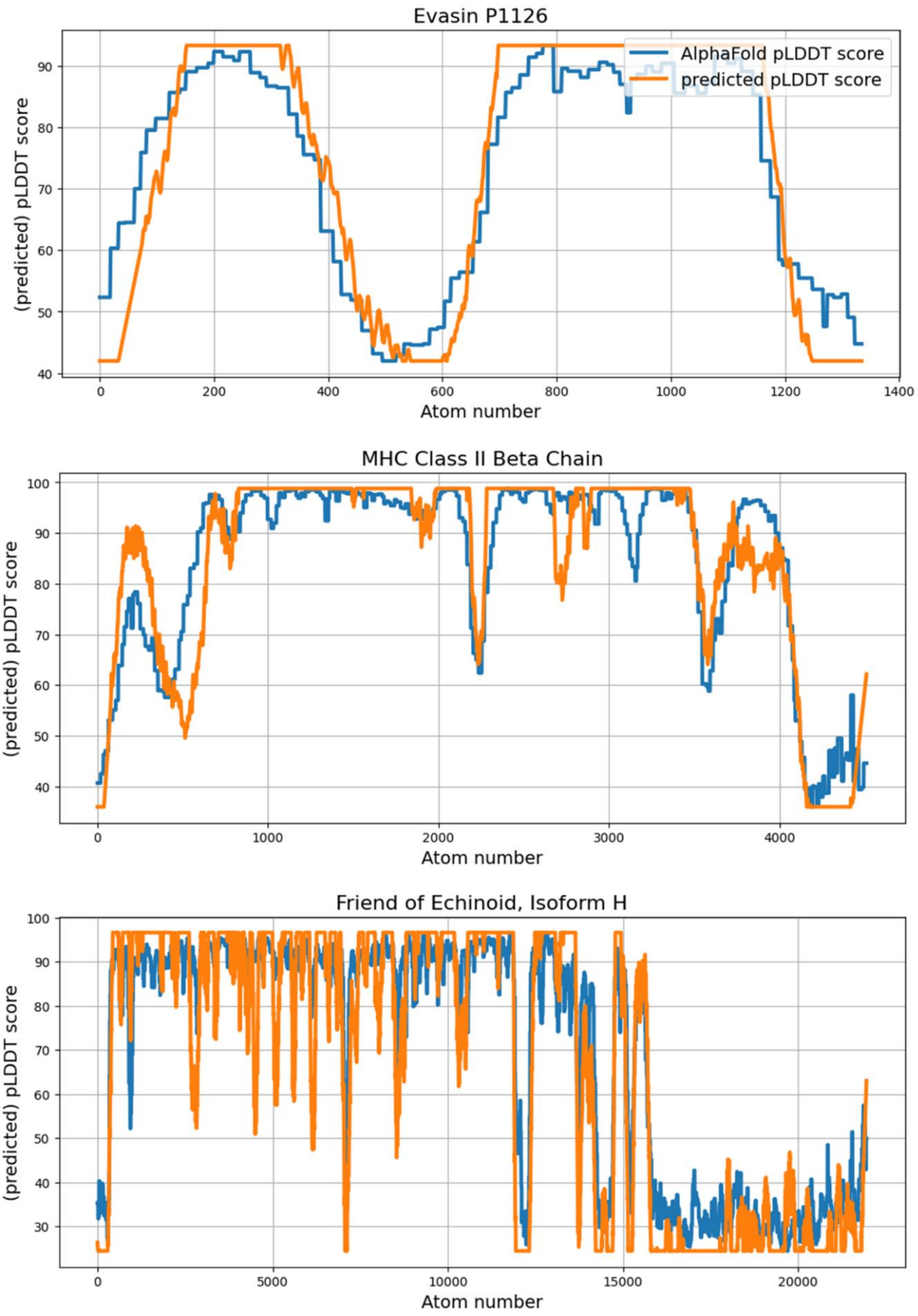

Supplementary Figure 12: AlphaFold pLDDT score in comparison with atomic energies. The pLDDT scores by AlphaFold (blue) and the predicted pLDDT scores computed with the described method using atomic energies (orange) are compared for the three predicted protein structures investigated in this work.

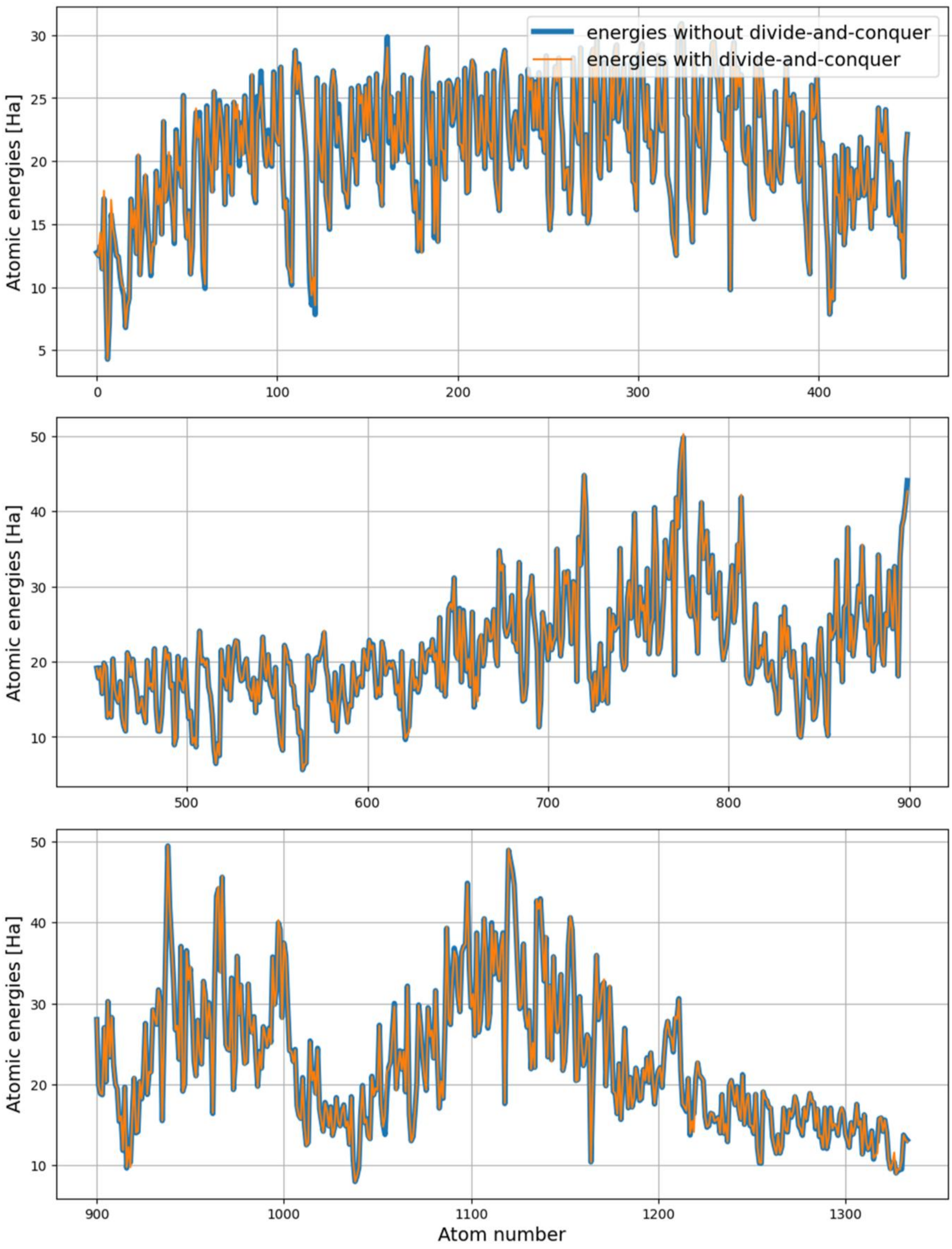

Supplementary Figure 13: Atomic energies with and without divide-and-conquer. Computed atomic energies are displayed for Evasin P1126, one of the AlphaFold predicted protein structures investigated in this work. The system was studied without water, otherwise a comparing calculation without divide-and-conquer would be computationally too demanding. The energies without using the divide-and-conquer method are plotted in blue and with a larger linewidth for a better visualization (to prevent too much overlap between the two lines) than the energies with divide-and-conquer in orange.

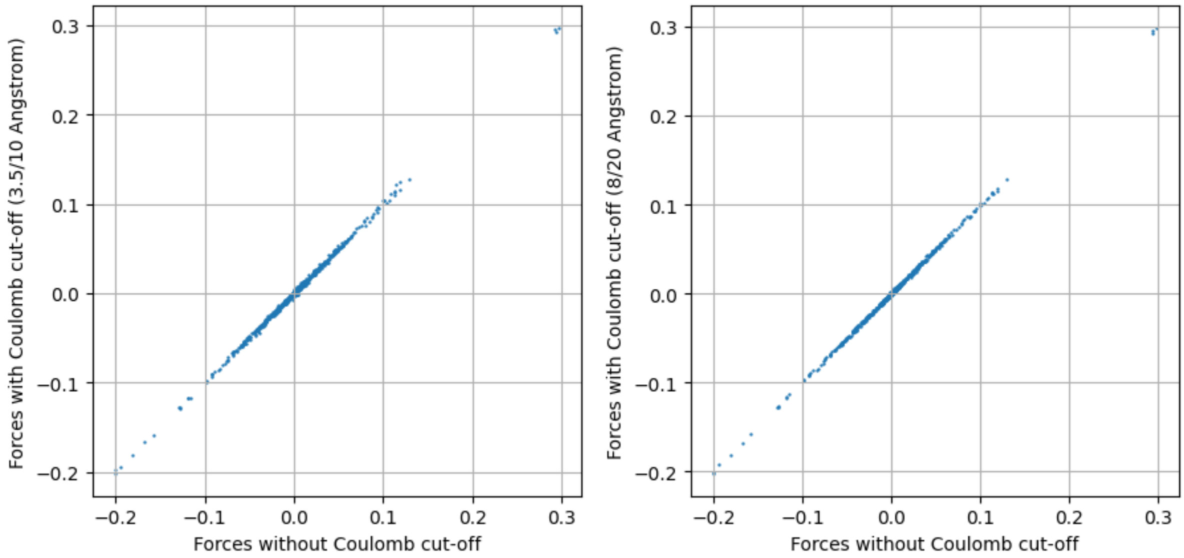

Supplementary Figure 14: Forces for DNA (ACGT) with and without Coulomb cut-off. For a DNA molecule (ACGT) the atomic forces are displayed without (x-axis) and with a Coulomb cut-off (y-axis) used. Two different Coulomb cut-offs configurations are tested for the lower and upper Coulomb cut-off used in this work. The mean absolute errors are 4.2 % and 2.5 %, respectively.

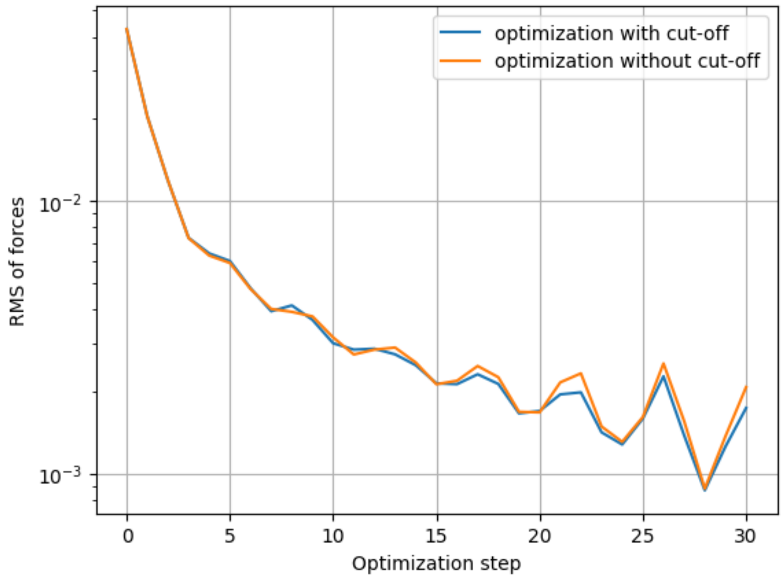

Supplementary Figure 15: Force RMS development under geometry optimization with and without cut-off for DNA (ACGT). A geometry optimization has been performed for a DNA tetramer (sequence ACGT) which uses the BFGS method with a corresponding golden section line search and ran over 30 optimization steps. The convergence to an equilibrium structure is evaluated by computing the root-mean-square of the computed forces at each optimization step.

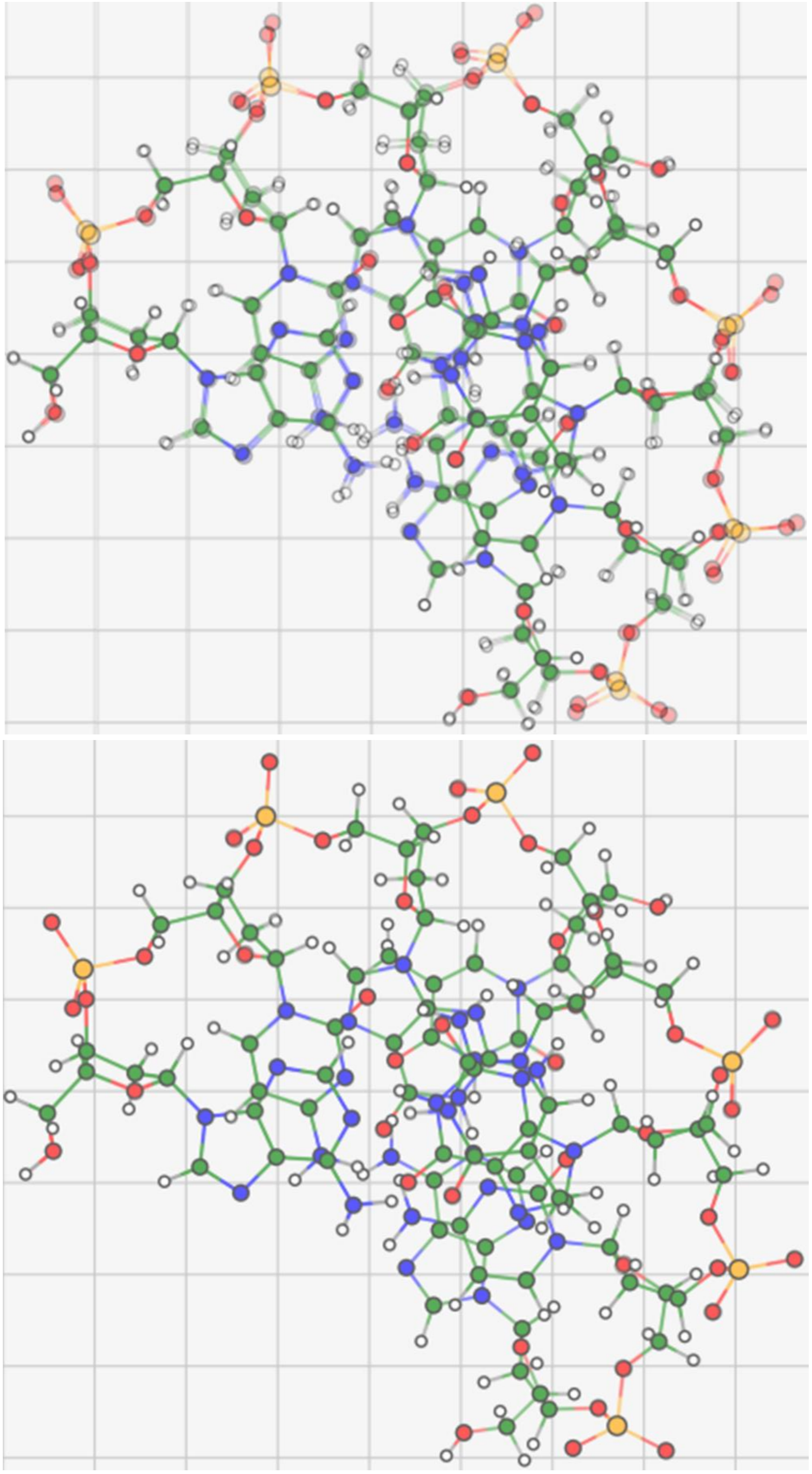

Supplementary Figure 16: Overlays of atomic coordinates from structural optimization. Top: The atomic coordinates from a DNA molecule (ACGT) from before the structural optimization are overlayed with the coordinates after the structural optimization with no Coulomb cut-off used. Bottom: The coordinates after structural optimization with no Coulomb cut-off used are overlayed with the coordinates after structural optimization with a Coulomb cut-off used with Coulomb cut-offs of 3.5 and 10 Angstrom for the lower and upper Coulomb cut-off, respectively.

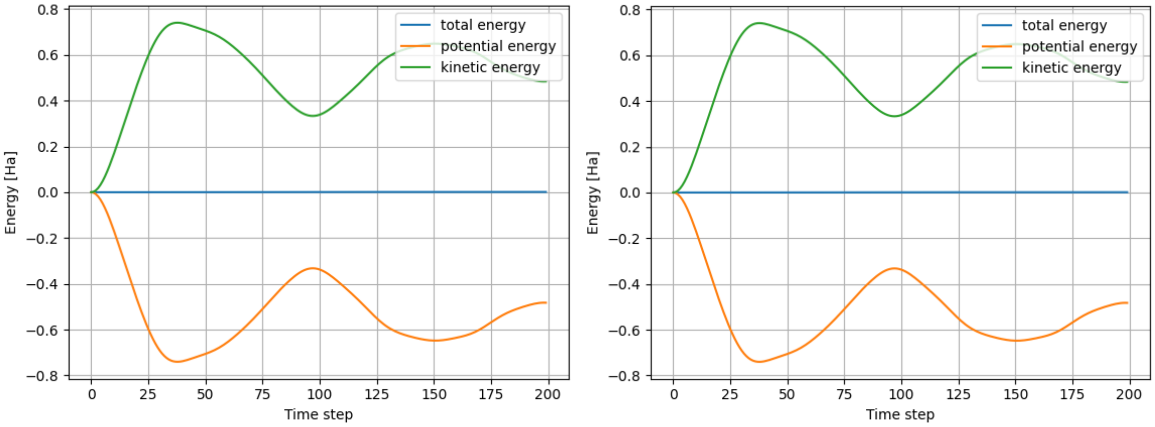

Supplementary Figure 17: Energy development during an MD simulation of DNA (ACGT) with and without cut-offs. A molecular dynamics simulation was performed for the DNA tetramer with the sequence ACGT. The development of the total, potential and kinetic energy is shown for the same simulation without (left) and with (right) a Coulomb cut-off used. For the simulation that uses a Coulomb cut-off, the values of the lower and upper Coulomb cut-off are 3.5 and 10 Angstrom respectively.

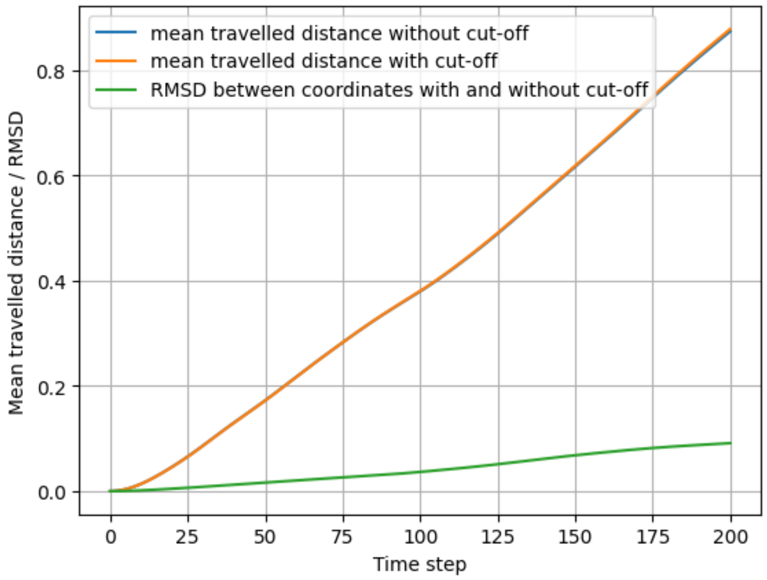

Supplementary Figure 18: Trajectory comparisons of MD simulation of DNA (ACGT) with and without cut-offs. For a molecular dynamics simulation of the mentioned DNA tetramer, the mean travelled distance is plotted for the same simulation with and without a Coulomb cut-off used. Additionally, the root-mean-square displacement between the coordinates of these two simulations is plotted for each time step. For the simulation that uses a Coulomb cut-off, the values of the lower and upper Coulomb cut-off are 3.5 and 10 Angstrom, respectively.

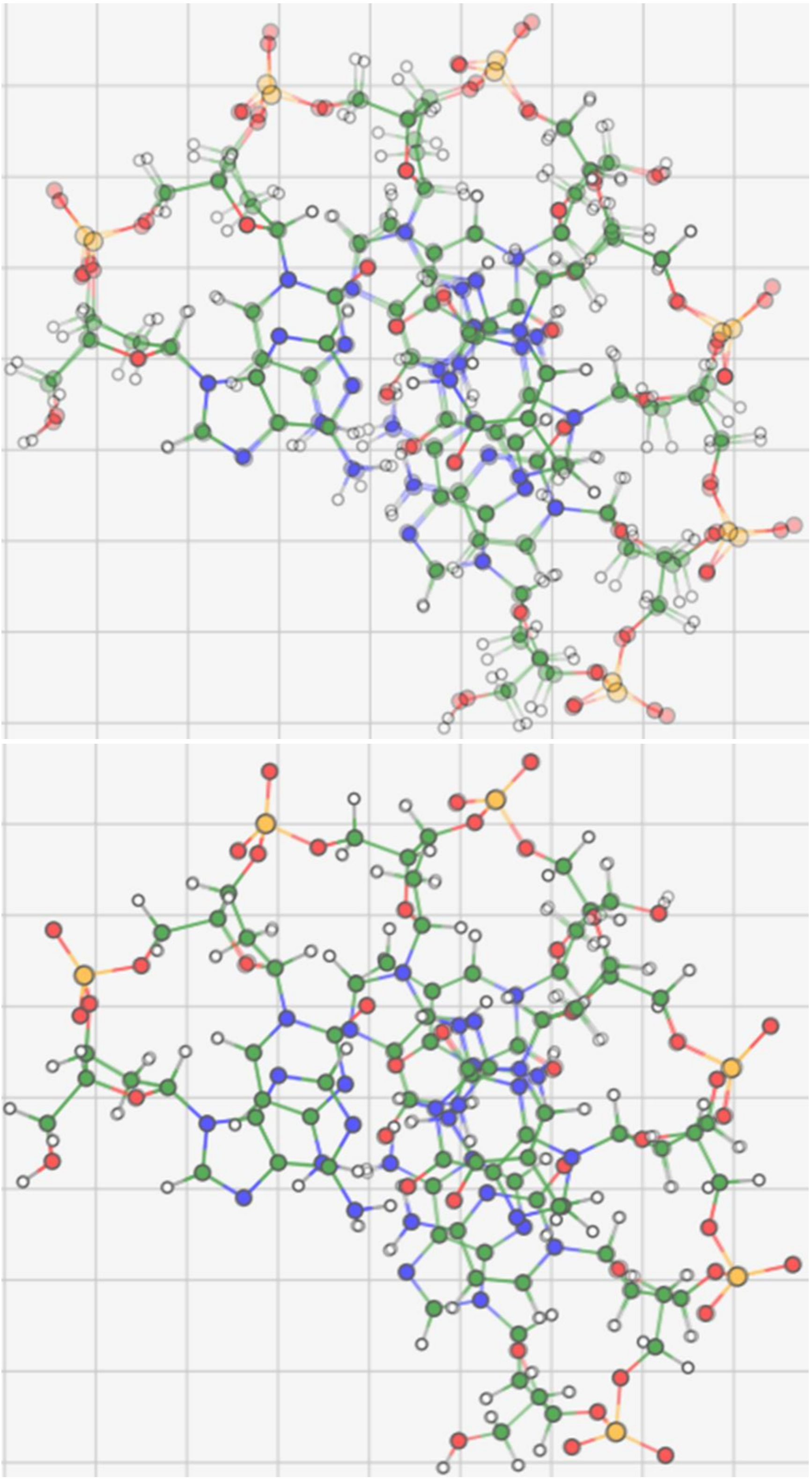

Supplementary Figure 19: Overlays of atomic coordinates from an MD simulation of DNA (ACGT). In a similar fashion to the results depicted in Figure 17, the coordinates are shown before and after an MD simulation. Top: Atomic coordinates from before and after an MD simulation of 200 time steps are overlayed. No Coulomb cut-offs were used in this calculation. Bottom: Overlay of the atomic coordinates after two MD simulations with identical starting conditions, one with Coulomb no cut-off and another one with Coulomb cut-off (3.5 and 10 Angstrom for the lower and upper cut-off).

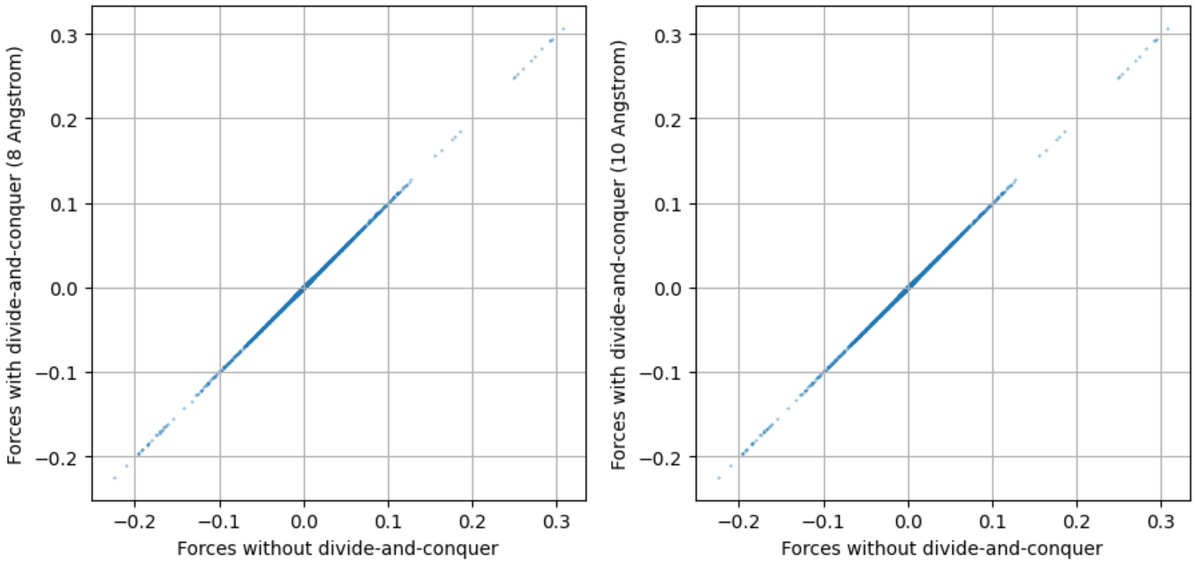

Supplementary Figure 20: Forces for DNA (ACGT) with and without divide-and-conquer. For a DNA molecule (ACGT) the atomic forces are displayed without (x-axis) and with the divide-and-conquer method (y-axis) used. Two different buffer zone configurations are tested (8 and 10 Angstrom). The mean absolute errors are 0.25 % and 0.047 %, respectively.

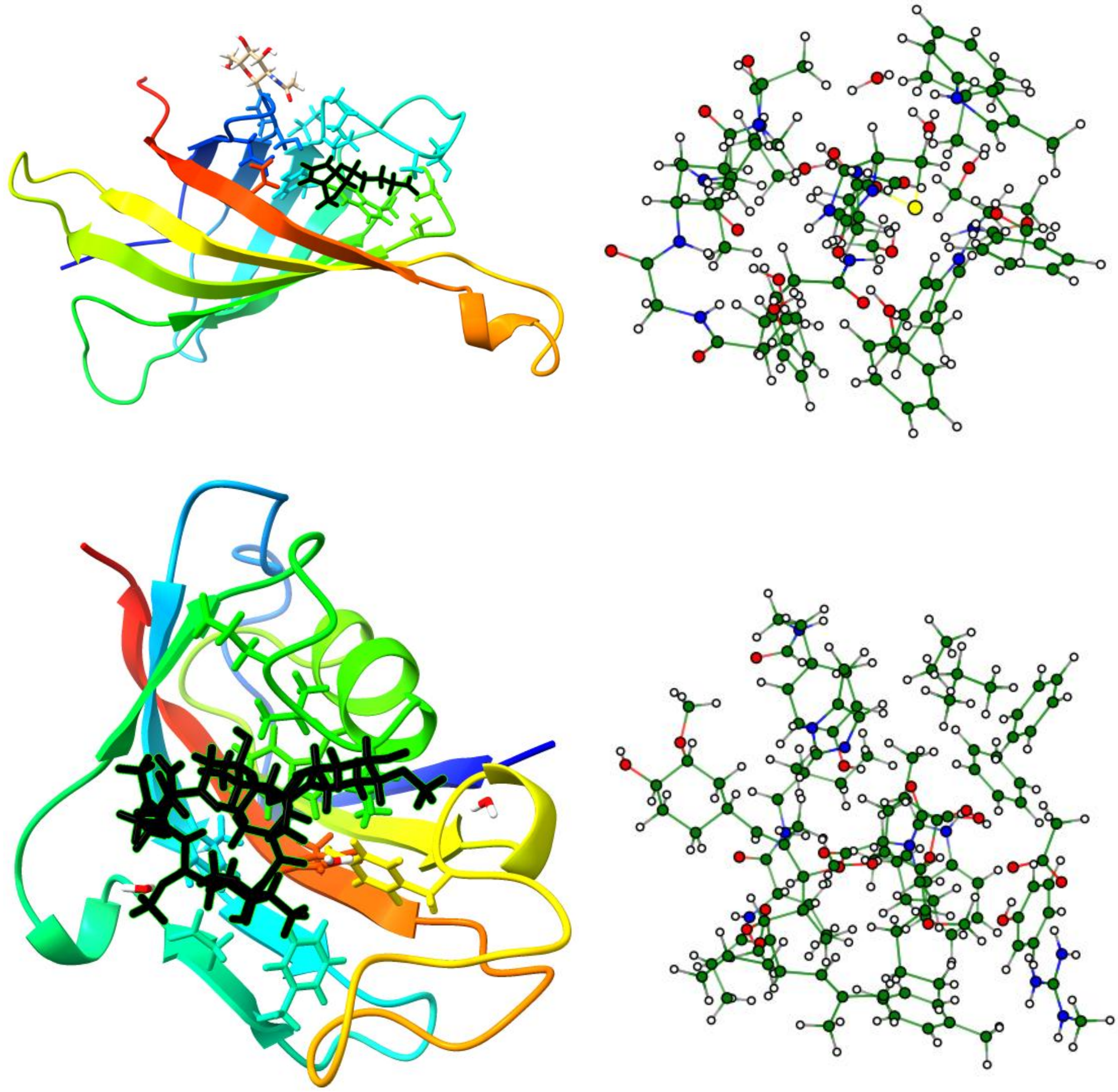

Supplementary Figure 21: Systems studied for binding energy tests. Two systems were used to compute the binding energy between a protein and a ligand. The first system (top row) is biotin bound to avidin (PDB 1AVD) and the second system (bottom row) is the drug FK506 bound to the protein FKBP (PDB 1FKJ). On the left side the protein is shown as a ribbon diagramme and the ligand is depicted in black. The ligand is studied in an environment of 4 Angstrom with neighbouring atoms added according to the procedure described in the corresponding section. The resulting structures for the two systems are shown on the right side.

**System**	**Density cut-off**	**Coulomb cut-off** [Angstrom]	**Computed binding energy** [kcal/mol]	**Experimental binding energy** [kcal/mol]
Biotin/Avidin	1.0e-6	None	–18.97	–20.4
Biotin/Avidin	1.0e-7	None	–19.23	–20.4
Biotin/Avidin	1.0e-8	None	–19.23	–20.4
Biotin/Avidin	1.0e-5	None	–28.07	–20.4
Biotin/Avidin	1.0e-6	3.5 / 10	–8.86	–20.4
Biotin/Avidin	1.0e-6	8 / 10	–41.86	–20.4
Biotin/Avidin	1.0e-6	15 / 20	–18.62	–20.4
FK506/FKBP	1.0e-6	None	–15.11	–12.8
FK506/FKBP	1.0e-7	None	–15.06	–12.8
FK506/FKBP	1.0e-8	None	–15.07	–12.8
FK506/FKBP	1.0e-5	None	–15.06	–12.8
FK506/FKBP	1.0e-6	3.5 / 10	0.96	–12.8
FK506/FKBP	1.0e-6	8 / 10	18.89	–12.8
FK506/FKBP	1.0e-6	15 / 20	–17.54	–12.8

Supplementary Table 1: Binding energies for two test systems for different cut-off configurations. For biotin bound to avidin (PDB 1AVD) and FK506 bound to FKBP (PDB 1FKJ) the binding energy is computed for different configurations.
